# Skin-directed Solius UVB light therapy increases vitamin D levels and alters intestinal microbiome and metabolome homeostasis

**DOI:** 10.3389/fmicb.2026.1850035

**Published:** 2026-08-12

**Authors:** Maira Jimenez-Sanchez, Hyungjun Yang, Ho Pan Sham, Zhiquan Clarence Huang, Jan P. Dutz, Bruce A. Vallance

**Affiliations:** 1 Department of Pediatrics, British Columbia (BC) Children's Hospital, University of British Columbia, Vancouver, BC, Canada; 2 Department of Dermatology and Skin Science, University of British Columbia, Vancouver, BC, Canada

**Keywords:** gut microbiota, gut-skin axis (GSA), metabolomics, UVB light therapy, vitamin D

## Abstract

The recent global rise in immune and inflammatory diseases, such as psoriasis and inflammatory bowel diseases, has been linked to Western society-based changes in lifestyle and environment. These include decreased exposure to sunlight in the ultraviolet B (UVB) spectrum, leading to impaired production of 25-hydroxyvitamin D. In addition, many of these diseases are linked to dysbiotic changes in gut microbiota composition and function. Clinical overlap between skin and gut diseases, along with growing evidence for a bidirectional “skin-gut axis,” supports the concept that exposing the skin to UVB light may impact the gastrointestinal tract including the microbiome. At present, exactly how UVB exposure influences gut microbial and metabolomic profiles and the role that baseline vitamin D status may play in these relationships remains unclear. To test this, we fed mice either a vitamin D sufficient or deficient diet and exposed them to Solius UVB light therapy (targeted peak of 293 nm) either acutely (single exposure) or repeatedly (six exposures) over 14 days. A single exposure modestly increased circulating vitamin D levels, whereas repeated exposures significantly increased levels to that seen in mice fed a vitamin D sufficient diet, and caused skin photoadaptation without histological injury. Notably, repeated Solius UVB light therapy induced significant changes in the composition of the gut microbiota, coinciding with marked fecal metabolome alterations, particularly in mice fed the vitamin D deficient diet. These fecal metabolic changes included increases in short chain fatty acids, as well as bile acid and tryptophan derivatives. In contrast, serum metabolomic responses were less marked, suggesting that Solius UVB light therapy induction of overt metabolic shifts within the intestinal tract is not merely reflective of systemic responses. Together, these findings demonstrate that skin-directed Solius UVB light therapy can be delivered safely, to induce significant changes in the composition of the gut microbiota and its associated metabolites, in a manner that depends on repeated exposures and baseline vitamin D status. Further studies exploring the ability of Solius UVB light therapy skin exposure to promote intestinal homeostasis and health are thus warranted.

## 1 Introduction

Sunlight is an important environmental signal that regulates both immune and hormonal responses (González Maglio et al., 2016). Modern lifestyles have significantly limited sun exposure through indoor living, sunscreen use, and sun avoidance, contributing to widespread vitamin D insufficiency (Holick, 2008). In fact, up to 47% of certain Westernized populations are considered vitamin D deficient (serum 25-hydroxyvitamin D (25(OH)D) level < 40 nmol/L ≈ 16 ng/mL), with the highest deficiency rates seen in individuals with darker skin pigmentation (Hyppönen and Power, 2007; Larose et al., 2014; Schwalfenberg et al., 2010). Serum 25(OH)D concentrations are expressed in nmol/L throughout, the internationally recommended SI unit; ng/mL equivalents are provided for clinical reference using the conversion factor 1 ng/mL = 2.5 nmol/L. Low vitamin D status (25(OH)D < 75 nmol/L = 30 ng/mL) has also been reported in up to 60–80% of individuals with inflammatory bowel disease (IBD) (Fletcher et al., 2019), as well as in patients with celiac disease (Stefano et al., 2023). In these conditions, vitamin D deficiency has been linked to impaired gastrointestinal (GI) mucosal barrier properties, dysregulated immune responses and increased susceptibility to inflammation and infection (Fletcher et al., 2019; Holick, 2008; Sun, 2010). Throughout this manuscript, “microbiota” refers to the community of gut-resident microorganisms (bacteria, archaea, fungi, and viruses), whereas “microbiome” refers to the microbiota together with their collective genetic repertoire and functional outputs, these terms are not used interchangeably (Hacioglu, 2026). These conditions are also associated with a dysbiotic gut microbiota, involving imbalances in both microbiota composition and function. To what degree these microbial imbalances reflect vitamin D insufficiency is currently unclear, but these findings do highlight the health risks associated with reduced sunlight exposure.

Amongst the entirety of the solar spectrum, ultraviolet B (UVB) radiation (290–320 nm) is the only stimulus capable of initiating cutaneous vitamin D synthesis (Leitenberger et al., 2007). UVB converts 7-dehydrocholesterol (7-DHC), a chromophore present in epidermal keratinocytes and dermal fibroblasts, into pre-vitamin D3, thereby initiating a pathway with well-established systemic immunological effects (Bikle, 2000). Beyond its role in vitamin D biology, UVB exposure also induces the formation of aryl hydrocarbon receptor (AhR) ligands in the skin (Memari et al., 2019). Cutaneous AhR signaling has been shown to regulate keratinocyte differentiation, epidermal barrier function, and local immune responses and has also been extensively characterized in the context of skin physiology and photobiology (Memari et al., 2019). The relevance of these UVB-induced cutaneous pathways to distal organs, such as the GI tract remains incompletely understood, however both vitamin D and AhR signaling are known to play critical roles in maintaining intestinal physiology (Assa et al., 2014; Fletcher et al., 2019; Postal et al., 2020).

Consistent with this conceptual framework, clinical observations indicate frequent overlap between skin and GI disorders. Chronic inflammatory skin diseases are frequently accompanied by GI symptoms such as diarrhea (Lundquist et al., 2025; Zysk et al., 2024), while intestinal diseases such as IBD commonly present with extraintestinal cutaneous manifestations (Huang et al., 2012). This clinical co-occurrence, together with the shared association with vitamin D deficiency and immune dysregulation, has contributed to increasing interest in studying interactions between the skin, gut, and immune system (De Pessemier et al., 2021; Jimenez-Sanchez et al., 2025). This has led to preclinical studies showing that skin-directed UVB exposure can lead to changes beyond the skin. In murine models, UVB skin exposure was shown to alter gut microbial community structure independent of dietary vitamin D intake (Ghaly et al., 2018). In humans, we previously showed that controlled UVB exposure in vitamin D-insufficient individuals leads to increased gut microbial diversity and changes in the relative abundance of bacteria known to produce different short-chain fatty acids (SCFA) (Bosman et al., 2019). However, the specific metabolic consequences of these microbiome changes, their temporal dynamics, and their dependence on baseline vitamin D status remain incompletely characterized.

Hypothesizing that UVB-induced cutaneous signals induce overt changes in gut-associated microbial and metabolic features (Dong and Perdew, 2020; Postal et al., 2020), we employed the Solius UVB light therapy system (with a targeted peak of 293 nm), a precisely defined wavelength range selected to approximate biologically relevant UVB exposure while minimizing DNA-damaging wavelengths. By combining controlled skin-directed UVB delivery with targeted dietary manipulation, longitudinal microbiome profiling, and paired stool and serum metabolomics, we demonstrate that exposure of the skin to Solius UVB light therapy leads to coordinated changes in gut microbial composition and metabolic profiles.

## 2 Methods

### 2.1 Mice and experimental diets

Weanling (3-week-old) female C57BL/6 mice were purchased from Charles River Laboratories (St. Constant, QC, Canada). Mice were fed either a vitamin D3-deficient diet [0 international units (IU)] or a vitamin D_3_-sufficient diet (1,000 IU/1,071.05 g diet) for 5 weeks, similar to previously described (Ghaly et al., 2018). All diets were purchased from Research Diets (New Brunswick, NJ, USA). Mice were maintained in sterilized, filter-topped cages, handled in tissue culture hoods, and given free-access to water under specific pathogen-free conditions in the animal facility at the BC Children's Hospital Research Institute. Sentinel animals were routinely tested for common pathogens. The protocols used were approved by the University of British Columbia's Animal Care Committee and in direct accordance with guidelines drafted by the Canadian Council on Animal Care.

### 2.2 Solius UVB light therapy protocol

Mice were anesthetized using 1% isoflurane combined with 1% O_2_. Approximately 8 cm^2^ of the dorsal fur of each mouse was shaved using a hair trimmer (Wahl Launch Trimmer), and the mice were then exposed to 250 mJ/cm^2^ of Solius UVB light therapy. The light source emits light with a targeted wavelength peak at 293 nm. Mice were exposed to the light source once to assess their acute Solius UVB light therapy response (with samples taken 24 h later), or repeatedly, at days 0, 2, 4, 7, 9, and 11 (with samples collected at day 14) to study the effects of repeated Solius UVB light therapy exposure. Control mice were shaved and handled in the same manner and frequency but were not exposed to Solius UVB light therapy.

### 2.3 Tissue and serum collection

Mice were anesthetized with isoflurane, and blood was collected by cardiac puncture. Blood was allowed to clot naturally at room temperature, the cells were removed by centrifugation, and then serum was collected and stored at −80 °C until analysis. Anesthetized mice were then euthanized by cervical dislocation, and skin tissue was collected from the dorsal region and immediately placed in 10% neutral buffered formalin (Fisher) for 24 h at 4 °C.

### 2.4 Histology

Skin samples were harvested, fixed in 10% neutral buffered formalin, dehydrated through a graded ethanol series, cleared in xylene, and embedded in paraffin. Paraffin-embedded skin tissues were sectioned at 4–5 μm thickness and mounted onto glass slides. Sections were deparaffinized in xylene and rehydrated through decreasing concentrations of ethanol to distilled water. Slides were stained with hematoxylin to visualize the overall tissue architecture, followed by rinsing in running tap water and bluing. Sections were then counterstained with eosin to visualize cytoplasmic components and extracellular matrix. After staining, slides were dehydrated through graded ethanol, cleared in xylene, and coverslips were added using a permanent mounting medium. Stained skin sections were imaged using brightfield microscopy to assess epidermal and dermal morphology.

### 2.5 Serum 25(OH)D3 analysis

Samples were prepared by combining 20 μL of plasma with labeled internal standards and precipitating serum protein using acetonitrile and methanol. The analyte was extracted using sodium chloride and hexane, followed by derivatization by PTAD as previously described (Ding et al., 2010) and performed by the Analytical Core for Metabolomics and Nutrition Core (RRID:SCR_026603). Samples and standards were analyzed by the Agilent 1290 Infinity II Bio LC coupled to the Agilent 6495D triple quadrupole mass spectrometer. The Waters Acquity BEH-C8 column (2.1 X 50 mm, 1.7 μm particle size) was used for chromatographic separation, using a binary gradient of 5 mM ammonium formate with 0.1% formic acid (v/v) and methanol.

### 2.6 Untargeted metabolomics analysis

Metabolome profiles of the sample extracts were acquired using flow-injection mass spectrometry by General Metabolics (Cambridge, MA, USA). The method used was adapted from Fuhrer et al., 2011. The instrumentation consisted of an Agilent 6550 iFunnel LC-MS Q-TOF mass spectrometer in tandem with an MPS3 autosampler (Gerstel) and an Agilent 1260 Infinity II quaternary pump. The running buffer was 60% isopropanol in water (v/v) buffered with 1 mM ammonium fluoride, using Hexakis (1H, 1H, 3H-tetrafluoropropoxy)-phosphazene) (Agilent) and 3-amino-1-propanesulfonic acid (HOT) (Sigma Aldrich). The isocratic flow rate was set to 0.150 mL/min. The instrument was run in 4GHz High Resolution, negative ionization mode. Mass spectra between 50 and 1,000 m/z were collected in profile mode. 5 uL of each sample were injected twice, consecutively, within 0.96 min to serve as technical replicates. The pooled study sample was injected periodically throughout the batch. Samples were acquired randomly within plates on the same instrument on the same day. Raw profile data were centroided, merged, and recalibrated using algorithms adapted from Fuhrer et al. (2011) (PMID: 21830798). Putative annotations were generated based on compounds contained in the Human Metabolome Database, KEGG, and ChEBI databases using both accurate mass per charge (tolerance 0.001 m/z) and isotopic correlation patterns.

### 2.7 Ingenuity pathway analysis (IPA)

Solius-UVB-light-therapy-responsive stool metabolites were identified based on false discovery rate (FDR)–corrected significance (*q* < 0.05) and absence of change in time-matched control samples. These Solius-UVB-light-therapy-specific metabolite lists were analyzed using Ingenuity Pathway Analysis (IPA, Qiagen) to infer associated disease and biofunction categories. Core analysis was performed using experimentally observed relationships curated in the IPA Knowledge Base, with enrichment significance calculated using Fisher's exact test. Disease and biofunction categories with an overlap *p* value < 0.05 were considered significantly enriched. Predicted functional activation or inhibition states were inferred using IPA activation Z-scores, which integrate the directionality of metabolite changes with curated causal relationships. Results were visualized using heatmaps displaying representative disease and biofunction categories, with color intensity corresponding to predicted activation (positive Z-scores) or inhibition (negative Z-scores). IPA-based predictions were used for hypothesis generation and interpretation of metabolomic signatures rather than as direct measures of functional activity.

### 2.8 Microbiota 16S rRNA gene sequencing and analysis

Fecal pellets were collected for 16S rRNA marker microbiota gene sequencing and analysis from control mice and mice given Solius UVB light therapy at day 0 (baseline before the first exposure) and 24 h after one Solius UVB light therapy exposure for cohort 1. For cohort 2, fecal pellets were collected at day 0 and day 14 (after six Solius UVB light therapy exposures). 16S rRNA library preparation for individual samples at Gut4Health (RRID: SCR_023673) was prepared similar to the method described in deWolfe and Wright (Wolfe and Wright, 2022). Briefly, the V4 region of the 16S rRNA gene was amplified with barcode primers containing the index sequences using a KAPA HiFi HotStart Real-time PCR Master Mix (Roche). PCR product amplification and concentration was monitored on a QuantStudio 3 Real-Time PCR system (Applied Biosystems). Amplicon libraries were then purified using AMPure XP Beads (Beckman), normalized based on concentration, and then pooled equally. Library concentrations were verified using a Qubit TM dsDNA high sensitivity assay kit (Invitrogen) and KAPA Library Quantification Kit (Roche) following manufacturer details. The purified pooled libraries were submitted to the Bioinformatics + Sequencing Consortium at UBC which verifies the DNA quality and quantity using an Agilent high sensitivity DNA kit (Agilent) on an Agilent 2100 Bioanalyzer. Sequencing was performed on the Illumina MiSeq TM v2 platform with 2 x 250 paired end-read chemistry at the UBC Sequencing + Bioinformatics Consortium.

### 2.9 16S rRNA gene amplicon processing and taxonomic profiling

Paired-end 16S rRNA gene amplicon sequencing data targeting the V3–V4 region (primers 341F/805R) were processed using QIIME2 (version 2024.2.0). Raw FASTQ files were imported using a paired-end manifest file in Phred33 format (PairedEndFastqManifestPhred33V2). Adapter sequences were removed using the QIIME2 *cutadapt* plugin, trimming Illumina TruSeq adapters from forward and reverse reads. Sequence quality and trimming performance were assessed using demultiplexing summaries to guide downstream filtering and denoising decisions. Samples with low sequencing depth were filtered using *qiime feature-table filter-samples*, retaining samples with a minimum total feature frequency of 10 reads. Features present in fewer than one sample were removed prior to denoising. Amplicon sequence variant (ASV) inference, quality filtering, paired-end merging, and chimera removal were performed using the DADA2 plugin (version 1.26.0). Reads were trimmed from the 5′ end by 17 bp (forward) and 21 bp (reverse), and truncated at 245 bp (forward) and 240 bp (reverse) based on quality score profiles, ensuring sufficient overlap across the ~425 bp V3–V4 amplicon. Maximum expected error thresholds were set to 2 (forward) and 3 (reverse). Taxonomic classification of ASVs was performed using a pre-trained Naive Bayes classifier based on the SILVA reference database (release 138.1), modified for the V3–V4 region and trained on sequences amplified with 341F/805R primers. Taxonomic composition was visualized using *qiime taxa barplot* with sample metadata.

### 2.10 Diversity metrics and differential abundance analyses

Alpha diversity was assessed using Shannon diversity and observed richness normalized per 1,000 reads, calculated in R (version 3.2.3) using the *vegan* package (version 2.5-2). Global differences in bacterial community composition were additionally visualized using weighted UniFrac distance metrics. Beta diversity was assessed using principal coordinates analysis (PCoA) based on Bray–Curtis dissimilarity to evaluate differences in microbial community composition among samples. Differential abundance analysis was performed using two complementary approaches. Analysis of Composition of Microbiomes with Bias Correction (ANCOM-BC) was applied to identify taxa differing significantly between experimental groups while accounting for compositionality and sampling bias. In parallel, DESeq2 was used to test for differential abundance using a negative binomial model with size-factor normalization. Results from both methods were compared to assess the robustness and consistency of Solius UVB light therapy associated microbial changes across time points and experimental cohorts.

### 2.11 Statistical analysis

Statistical analyses were performed using R (version 2023.06.0+421) and Python. Depending on data distribution, statistical significances were assessed using either a two-tailed Student's *t*-test or the Mann–Whitney U test, unless otherwise indicated. For comparisons involving multiple groups, one-way ANOVA or the Kruskal–Wallis test with appropriate *post-hoc* analyses was applied. For metabolomics analyses, raw intensity values were processed in Python. Prior to statistical testing, outliers were removed using an interquartile range (IQR)-based filtering approach, where values falling outside the range [*Q*1 − 1.5 × *IQR, Q*3 + 1.5 × *IQR*] were excluded on a per-metabolite basis. Only metabolites with at least two valid observations per group after filtering were retained for statistical testing. Differential metabolite abundance between groups was assessed using Welch's two-sample *t*-test (unequal variance). Log2 fold change (log2FC) was calculated based on the ratio of group means, with a small constant added where necessary to avoid division by zero. Resulting *p*-values were adjusted for multiple comparisons using the Benjamini–Hochberg false discovery rate (FDR) correction. Metabolites with an adjusted *q*-value < 0.05 were considered statistically significant.

β-diversity was calculated using Bray–Curtis dissimilarity, and differences in microbial community composition were assessed using permutational multivariate analysis of variance (PERMANOVA; *adonis2*) with 999 permutations, with the random seed set to 123 for reproducibility. PERMANOVA analyses were performed within Treatment × Diet strata to evaluate temporal effects across time points. Following identification of significantly altered stool metabolites, pathway enrichment analysis was performed using MetaboAnalyst 6.0 with the Gut Microbiota + Host KEGG pathway library.

To assess integrative microbiome–metabolite relationships, Spearman rank correlation analyses (ρ) were conducted between delta-transformed microbial genus abundances and delta-transformed metabolite levels. Correlation significance was assessed using two-tailed tests, and results are reported as correlation coefficients (ρ) with corresponding *P* values. Statistical significance was defined as *P* < 0.05.

### 2.12 Data visualization

Data visualization and graphical representation were performed in Python using the *pandas, NumPy, seaborn*, and *matplotlib* libraries. Volcano plots were generated to visualize differential metabolite abundance and statistical significance. Correlation heatmaps were generated with color intensity representing Spearman correlation coefficients (ρ) and overlaid text indicating corresponding *P* values.

## 3 Results

Solius UVB light therapy induces skin photoadaptation without histologic injury in concert with elevated circulating vitamin D levels

**Figure 1:**
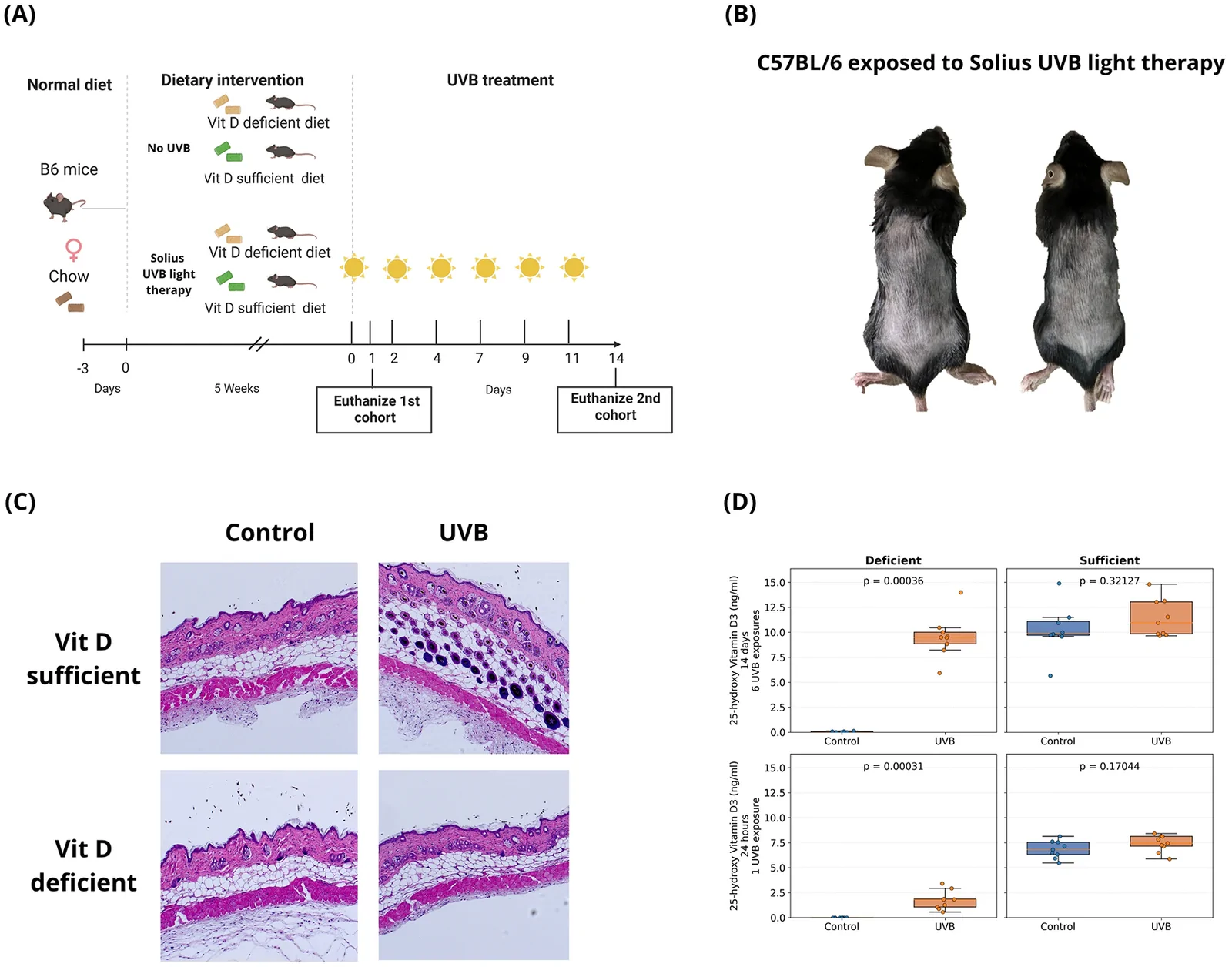
Solius UVB light therapy exposure induces skin photoadaptation and increases circulating vitamin D levels in mice. **(A)** Experimental design and timeline. Female C57BL/6 mice were maintained on vitamin D-sufficient or vitamin D-deficient diets for five weeks prior to Solius UVB light therapy exposure. Mice then received Solius UVB light therapy or no Solius UVB light therapy, followed by either a single exposure (acute cohort) or six repeated exposures over 14 days (chronic cohort). Animals were euthanized 24 h after the final exposure for downstream analyses. **(B)** Representative dorsal view of shaved mice illustrating the uniform Solius UVB light therapy exposure area used throughout the study. **(C)** Representative H&E-stained dorsal skin sections (10X) from vitamin D-sufficient and vitamin D-deficient mice exposed to six sessions of Solius UVB light therapy or left untreated. Representative H&E-stained skin sections obtained after six Solius UVB light therapy exposures demonstrate preserved epidermal architecture and features consistent with physiological photoadaptation, with no evidence of epidermal disruption, necrosis, or inflammatory damage. Vitamin D–deficient mice display marked epidermal hyperplasia, defined by expansion of viable epidermal layers and increased stratum corneum thickness. Images are representative of each experimental group. **(D)** Serum 25-hydroxyvitamin D3 [25(OH)D3] concentrations measured 24 h after a single Solius UVB light therapy exposure (bottom panels) or after six repeated exposures (top panels) in vitamin D-deficient and vitamin D-sufficient mice. Solius UVB light therapy exposure induced a marked increase in circulating 25(OH)D3 in vitamin D-deficient mice after both acute and repeated exposure, reaching levels comparable to vitamin D-sufficient controls following chronic Solius UVB light therapy treatment. In contrast, vitamin D-sufficient mice showed modest or non-significant changes. Individual data points are shown with box-and-whisker plots. Statistical significance was assessed using Mann–Whitney tests, with exact *p*-values indicated.

To investigate whether controlled skin-directed Solius UVB light therapy exposure can exert systemic effects beyond vitamin D synthesis, such as altering gut microbiota composition and function, we first established the efficacy and safety of the Solius UVB light therapy device, and its ability to increase vitamin D levels. Mice fed a vitamin D sufficient diet, or a vitamin D deficient diet for five weeks, received either one dose of Solius UVB light therapy (cohort 1), or six doses (cohort 2) as shown in Figure 1A. No visible macroscopic changes in the skin were seen in any group as shown in Figure 1B.

We next histologically examined exposed skin tissues to assess the cutaneous reactions to Solius UVB light therapy exposure. No overt histological changes were noted after one exposure, whereas after six exposures, the dorsal skin of both vitamin D sufficient and vitamin D deficient mice exposed to 250 mJ/cm^2^ Solius UVB light therapy exhibited photoadaptive histological changes (Figure 1C). Skin sections from control mice (untreated) under vitamin D sufficient and deficient diets were comparable, showing a layered epidermis with a thin and compact stratum corneum. In vitamin D sufficient Solius-UVB-light-therapy-treated mice, modest epidermal hyperplasia was observed, with normal epidermal layering. Vitamin D deficient Solius-UVB-light-therapy-treated mice showed greater epidermal hyperplasia characterized by an increase in the thickness of the viable epidermis and in stratum corneum thickness, with irregularities in the stratification of the epidermis. While suggesting a greater epidermal proliferative response in the vitamin D deficient mice, the modest histologic responses seen in exposed mice suggests that both groups developed the expected physiologic photoadaptation to Solius UVB light therapy. There was no evidence of epidermal destruction, necrosis or inflammation, as compared to prior studies using different UVB wavelengths (Savic et al., 2019). These findings confirm the safety of the Solius UVB light therapy protocol used in this model.

To test whether these sessions of Solius UVB light therapy elicited systemic vitamin D responses, we quantified serum calcifediol (i.e., 25-hydroxyvitamin D_3_) levels at 24 h after a single Solius UVB light therapy exposure and also at 14 days after six exposures (Figure 1D). Although baseline levels of calcifediol were undetectable in mice fed the vitamin D deficient diet, one exposure to Solius UVB light therapy significantly increased serum calcifediol levels (1.73 ± 0.90 ng/ml) in deficient animals, thus demonstrating both rapid and efficient synthesis of vitamin D. Importantly, six exposures to Solius UVB light therapy further, and significantly increased circulating calcifediol concentrations to levels similar to those observed for vitamin D sufficient animals (Vitamin D sufficient Control: 10.25 ± 2.70 ng/ml vs. Vitamin D deficient + Solius UVB light therapy: 9.55 ± 2.30 ng/ml).

These results demonstrate that exposure to the Solius UVB light therapy device results in physiological photoadaptation of the skin and rapid increases in serum vitamin D levels without causing overt histological damage. This system thus provides a well-controlled platform to assess whether skin-directed Solius UVB light therapy can induce downstream effects in remote organs such as the GI tract.

### 3.1 Repeated Solius UVB light therapy remodels gut microbiota composition over time

**Figure 2:**
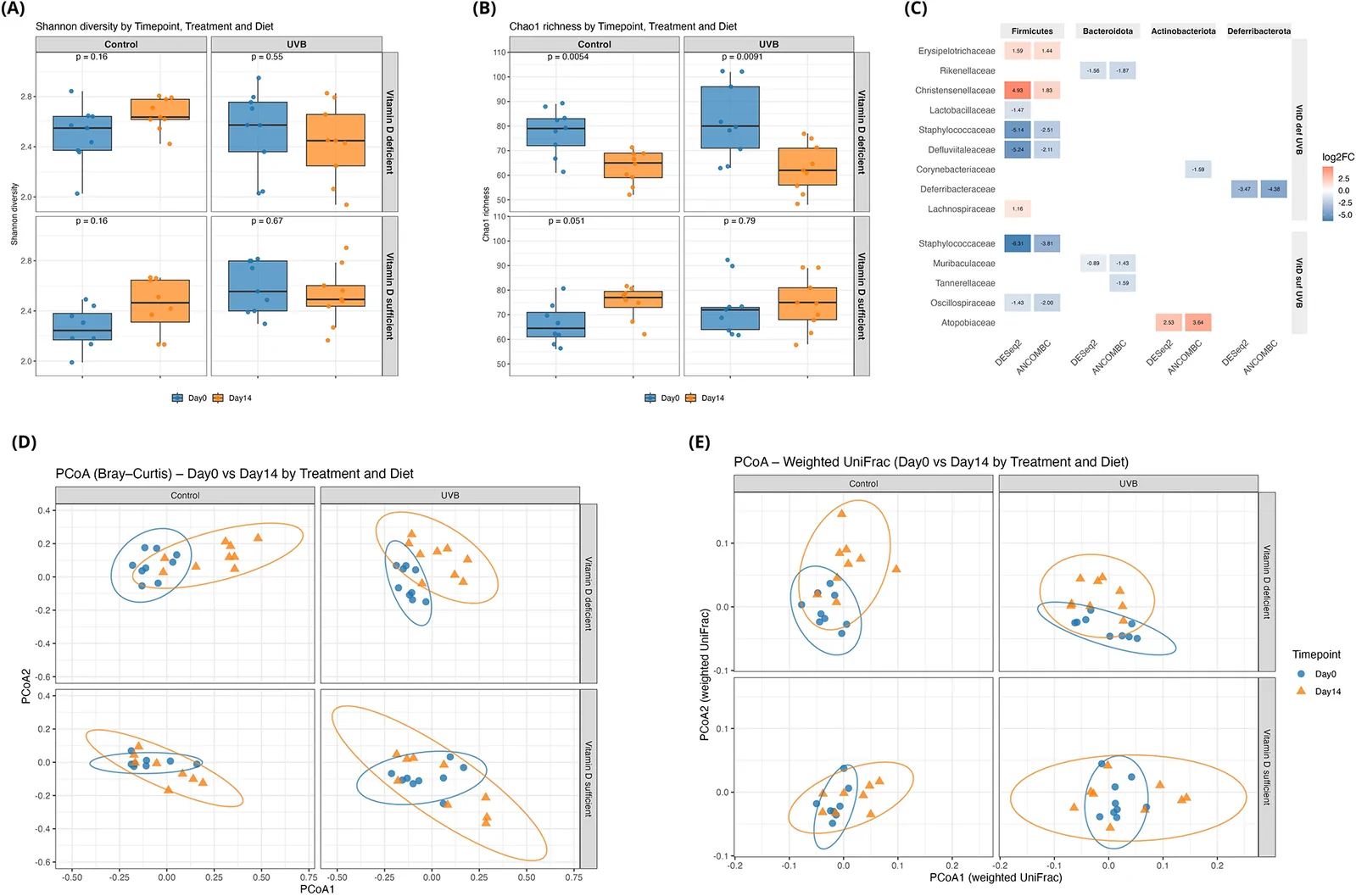
Microbiota analysis**. (A, B)** Alpha-diversity metrics at Day 0 and Day 14 across diet and treatment groups. Chao1 richness and Shannon diversity were largely stable over time, with modest richness increases in selected groups. **(C)** Bray–Curtis PCoA plots illustrating compositional separation between Day 0 and Day 14 communities within each diet–treatment combination (PERMANOVA *R*^2^ = 0.20–0.36, *p* ≤ 0.021). **(D)** Weighted UniFrac PCoA incorporating phylogenetic distance. (Vitamin D deficient Control: *R*^2^ = 0.40, *p* = 0.002; Solius UVB light therapy: *R*^2^ = 0.19, *p* = 0.026; vitamin D sufficient Control: *R*^2^ = 0.15, *p* = 0.05; Solius UVB light therapy: *R*^2^ = 0.05, *p* = 0.4). **(E)** Differential-abundance summary (ANCOM-BC and DESeq2) showing log_2_ fold-changes in bacterial families from Day 0 to Day 14 in Solius-UVB-light-therapy-treated mice. Full results for controls are shown in Supplementary Figure 2.

As we found that repeated Solius UVB light therapy was well tolerated and significantly increased serum vitamin D concentrations, we next examined whether this intervention was associated with changes in the gut microbiota, as previous studies have shown that vitamin D can modulate microbial composition as well as host–microbe interactions (Bashir et al., 2016; Cantorna et al., 2014). We collected feces from mice of both diet groups, at day 0, as well as on day14, after six exposures to Solius UVB light therapy. As shown in Figure 2A, Shannon diversity remained stable amongst diet and treatment groups, whereas Chao1 richness was significantly reduced in vitamin D deficient mice, but this decrease was observed in both control and Solius UVB light therapy exposed animals, indicating that time, rather than Solius UVB light therapy exposure was involved (Figure 2B). In vitamin D sufficient mice, Chao1 richness tended to increase whereas Shannon diversity remained stable among diets and treatments (Figure 2B). These trends suggest that UVB exposure does not result in a net loss or increase in microbial **α**-diversity, however slight shifts in the richness component may occur.

Meanwhile, a clear restructuring of microbial community composition in response to Solius UVB light therapy was shown by β-diversity analyses. Bray–Curtis PCoA revealed a significant temporal distance in community composition between timepoints Day 0 and Day 14 for all combinations of diet-treatment (PERMANOVA *R*^2^ = 0.20–0.36, *p* ≤ 0.021; Figure 2C). When integrating phylogenetic relationships (using weighted UniFrac) temporal-related effects were more pronounced under conditions of vitamin D deficiency (Control: *R*^2^ = 0.40, *p* = 0.002; UVB: *R*^2^ = 0.19, *p* = 0.026) whereas microbiota shifts in vitamin D sufficient mice were weaker or insignificant (Control: *R*^2^ = 0.15, *p* = 0.05; UVB: *R*^2^ = 0.05, *p* = 0.4; Figure 2D). These findings suggest that the structure of the gut microbiota changes over time, and that UVB exposure as well as vitamin D status influence the trajectory.

To identify the individual taxa that drove these changes, we performed differential-abundance testing with two techniques: ANCOM-BC (compositional model) and DESeq2 (count-based negative-binomial model). Figure 2E shows a summary of Solius UVB light therapy associated log_2_ fold changes at Day 14, as compared to Day 0 for the Solius UVB light therapy treated groups. The control comparisons are shown in Supplementary Figure 1. Specifically, in the Solius-UVB-light-therapy-irradiated vitamin D deficient mice, a number of bacterial families significantly increased in abundance including *Erysipelotrichaceae, Christensenellaceae*, and *Lachnospiraceae*. These families have been previously associated with changes in lipid metabolism (Kaakoush, 2015) and SCFA production (Singh et al., 2023; Waters and Ley, 2019). At the same time, repeated Solius UVB light therapy exposures were coupled with decreases in families frequently enriched under inflammatory or dysbiotic gut conditions, including *Staphylococcaceae, Corynebacteriaceae* and *Deferribacteraceae*. Other bacterial families that underwent changes in abundance, such as *Rikenellaceae* and *Defluviitaleaceae*, were also diet- and host-dependent, although their impact on host functions are as yet, poorly defined (Tavella et al., 2021). Reductions in the *Lactobacillaceae* family were also noted, with at least some members of this family considered beneficial (Huynh and Zastrow, 2023).

As compared with untreated controls under a vitamin D deficient diet, some families that increased in Solius UVB light therapy treated mice (*Erysipelotrichaceae* and *Lachnospiraceae*) also rose over time in untreated controls (a background temporal effect). In contrast, expansion of *Christensenellaceae* was stronger under Solius UVB light therapy, indicating a UVB-specific effect. Importantly, the *Christensenellaceae* family has been associated with leanness and protection against obesity in many previous studies (Waters and Ley, 2019). Moreover, Solius UVB light therapy resulted in a significant loss in abundance of the *Deferribacteraceae* which was supported by both DESeq2 and ANCOM-BC analysis. Although a reduction was seen in the control mice, this observation was only detected using DESeq2. In addition, the agreement in results between Solius-UVB-light-therapy-treated mice using either method suggests a more consistent effect of Solius UVB light therapy exposure. In contrast, the signal found in the control group could reflect variations due to assay-specific sensitivities or changes in composition, rather than a stable biological change.

Solius UVB light therapy also induced a unique compositional signature in vitamin D sufficient mice, leading to a relative increase in abundance of *Atopobiaceae* and decreases in *Muribaculaceae, Tannerellaceae, Staphylococcaceae*, and *Oscillospiraceae*. Functionally, these microbes are often associated with carbohydrate fermentation [*Atopobiaceae* (Morinaga et al., 2022), *Muribaculaceae* (Zhu et al., 2024)], mucin and host-glycan utilization [*Tannerellaceae* (Wexler, 2007)], and immune interaction or dysbiosis-linked expansion (*Staphylococcaceae* [Thursby and Juge, 2017)]. *Oscillospiraceae* family members often have the capacity to produce butyrate, so their reduction could prove detrimental (Elford et al., 2024).

These results demonstrate that repeated Solius UVB light therapy in mice remodels the structure of gut microbial communities, without causing major losses in diversity. Importantly, amplified Solius UVB light therapy associated shifts in microbial composition, suggesting that baseline vitamin D status influences how microbial communities respond over time to skin-directed Solius UVB light therapy. As a result, Solius UVB light therapy may influence the microbial composition not only indirectly through vitamin D-related pathways but also via other systemic cues.

### 3.2 Solius UVB light therapy induces time-dependent and vitamin-d–sensitive remodeling of gut metabolome

Having established that Solius UVB light therapy alters gut microbial composition over time, we next examined whether these alterations impacted the intestinal metabolome. We initially focused on stool metabolomic profiles at day 14 (after six exposures) to represent the cumulative impact of repeated Solius UVB light therapy exposure. We also evaluated any metabolomic responses apparent 24 h after only one Solius UVB light therapy exposure (Day 1) to determine if these early metabolic reactions antecede and/or differ from those seen at day 14.

#### 3.2.1 Broad remodeling after 14 days of Solius UVB light therapy - driven predominantly by vitamin-D deficiency

**Figure 3:**
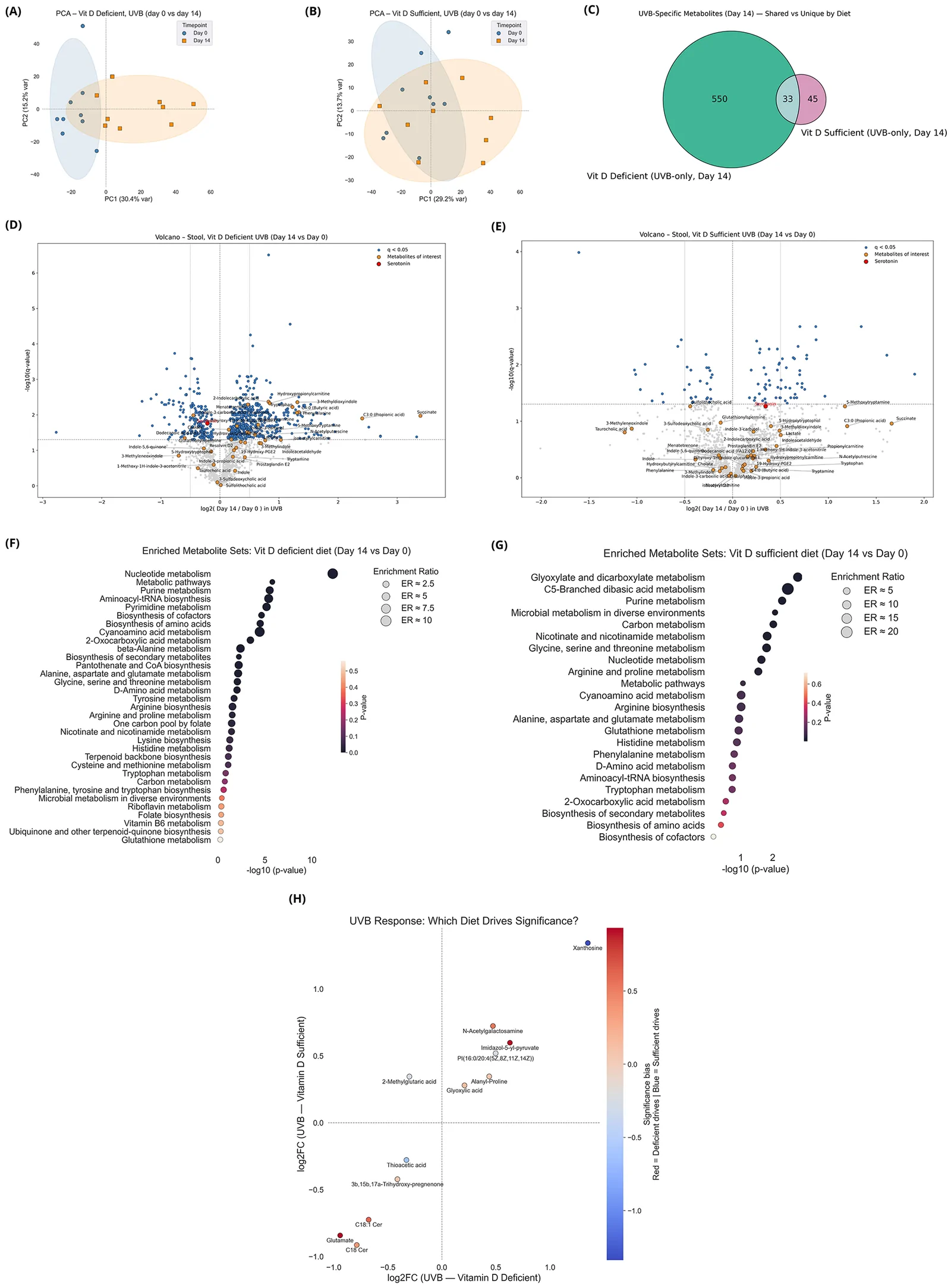
Metabolomic analysis. **(A)** PCA of stool metabolomes from vitamin D–deficient mice at Day 0 (blue) and Day 14 (orange) following Solius UVB light therapy shows a marked temporal shift (PERMANOVA: pseudo-*F* = 4.24, *p* = 0.001; 95% ellipses). **(B)** PCA of vitamin D–sufficient mice at Day 0 and Day 14 reveals a significant but more modest separation (PERMANOVA: pseudo-F = 2.87, *p* = 0.007; 95% ellipses). **(C)** Venn diagram of Solius-UVB-light-therapy-specific stool metabolites at Day 14 (FDR *q* < 0.05), defined as metabolites altered by Solius UVB light therapy but unchanged in matched controls, highlighting a predominance of diet-specific responses. **(D, E)** Volcano plots of Solius-UVB-light-therapy-induced metabolite changes at Day 14 vs. Day 0 in vitamin D–deficient **(D)** and vitamin D–sufficient **(E)** mice. **(F, G)** Pathway enrichment of Solius-UVB-light-therapy-responsive metabolites at Day 14 in vitamin D–deficient **(F)** and vitamin D–sufficient **(G)** mice (MetaboAnalyst; Gut Microbiota + Host KEGG). Bubble size reflects enrichment ratio and color indicates –log10(p). Xenobiotic-like annotations were excluded. **(H)** Agreement analysis of Solius-UVB-light-therapy-responsive metabolites at Day 14 showing relative statistical support in deficient vs. sufficient mice.

On Day 14 after six Solius UVB light therapy exposures, the patterns of stool metabolomic profiles were clearly altered as compared to baseline. Principal component analysis (PCA) showed distinct clustering of Day 0 samples as compared to the Day 14 samples for both diet groups (Figures 3A, B) and this visual trend was validated by statistical analysis. In vitamin D-sufficient mice, metabolic changes were significant in Solius-UVB-light-therapy-treated animals (pseudo-F = 2.87, *p* = 0.007) and control animals (pseudo-F = 2.28, *p* = 0.005, Supplementary Figure 2B). Vitamin-D-deficient mice also displayed pronounced temporal dissociation in Solius-UVB-light-therapy-treated (pseudo-*F* = 4.24, *p* = 0.001) and control mice (pseudo*-F* = 2.39, *p* = 0.008, Supplementary Figure 2A). While some of observed metabolic changes could be attributed to time, effect sizes were always larger in Solius-UVB-light-therapy-exposed mice, especially for the vitamin D deficient mouse group.

Focusing on those following Solius UVB light therapy but were constant across all controls (FDR q > 0.05), we identified far more Solius-UVB-light-therapy-specific responsive metabolites in the vitamin D deficient group, i.e., 550 Solius-UVB-light-therapy-unique vs. the 45 shown in the vitamin D sufficient group. Notably, 33 metabolites were altered in common between the two diet groups (Figure 3C) and most of the Solius UVB light therapy responsive metabolites showed time-dependent increases.

Interestingly, a large proportion of the Solius-UVB-light-therapy-altered metabolites are either produced by microbes or are linked to host-microbiota interactions that generally promote gut health. For example, Solius UVB light therapy increased the abundance of the propionic and butyric acids (C3:0, C4:0) in both diet groups, as shown in Figures 3D, E. UVB exposures also increased levels of succinate, lactate, and SCFA-derived acylcarnitines, reflecting increased microbial fermentation. Polyamine intermediates (N-acetyl- and γ-glutamyl-putrescine) as well as metabolites associated with mucin and bacterial cell-wall remodeling were also increased, in concert with changes in specific bile-acids suggesting active crosstalk between the microbiota and its host.

Pathway enrichment analysis (MetaboAnalyst 6.0, Gut Bacteria and Host KEGG library) of vitamin D deficient mice (Figure 3F) highlighted pathways related to amino-acid and cofactor biosynthesis (panthothenate/CoA, folate, riboflavin, vitamin B6), nucleotide metabolism, one-carbon processing and several nitrogen-processing modules. Moreover, enrichment analysis indicated alterations in tryptophan metabolism with significant changes in tryptophan, 5-methoxy-tryptamine and tryptophan-containing dipeptides (Figures 3D, E). Fewer, and potentially more selective metabolic responses to Solius UVB light therapy were seen in vitamin D-sufficient mice (Figure 3E). Those that achieved significance (Figure 3G) included anabolic/nucleotide pathways, such as amino-acid biosynthesis, glyoxylate/dicarboxylate metabolism, and nicotinamide/NAD metabolism, and purine metabolism. Interestingly, serotonin levels were also increased in vitamin D sufficient mice and approached statistical significance (*q* ≈ 0.05), whereas serotonin levels decreased under vitamin D deficient conditions (Figures 3D, E). As the gut supplies 90–95% of the body's serotonin (Liu et al., 2021), which plays an influential role in intestinal motility, secretion, epithelial function and gut-brain signaling, these divergent results suggest that Solius UVB light therapy exposure can modulate the tryptophan/serotonin axis, in a manner dependent on baseline vitamin D status.

To compare between diets and determine the robustness of the overlap in shared Solius UVB light therapy responsive metabolites, they were analyzed by a significance-bias index (–log10 qDEF – –log10 qSUF) (Figure 3H). This approach enabled direct comparison of which vitamin D diet most strongly supported statistical significance for the same Solius-UVB-light-therapy-responsive features. The majority of metabolites clustered close to neutrality with a slight shift toward vitamin D deficiency, most notably among glutamate- and indole-related molecules, whereas nucleoside-linked compounds such as xanthosine were following Solius UVB light therapy of vitamin D sufficient mice. Thus, Solius-UVB-light-therapy-driven remodeling of the intestinal metabolome is influenced by vitamin D deficiency. The enrichment of fermentation products, mucosal-associated metabolites, and tryptophan-derived compounds suggests that Solius UVB light therapy promotes coordinated metabolic adaptation within the gut ecosystem rather than transient perturbations.

#### 3.2.2 Early effects (Day 1): rapid but diet-dependent Solius-UVB-light-therapy-driven stool metabolic shifts

To determine how rapidly Solius UVB light therapy induces metabolic responses, we next compared stool metabolomic profiles between baseline (day 0) and 24 hours post-exposure for each treatment group. A 24-h post-Solius-UVB-light-therapy time point was selected, given that previous studies in murine models have shown that a single Solius UVB light therapy induces changes in the skin metabolomic profile within hours (Patra et al., 2023). Moreover, studies on molecular responses of human skin cells suggest that significant cellular alterations are evident by 24 h after the exposure (Khalil and Shebaby, 2017).

**Figure 4:**
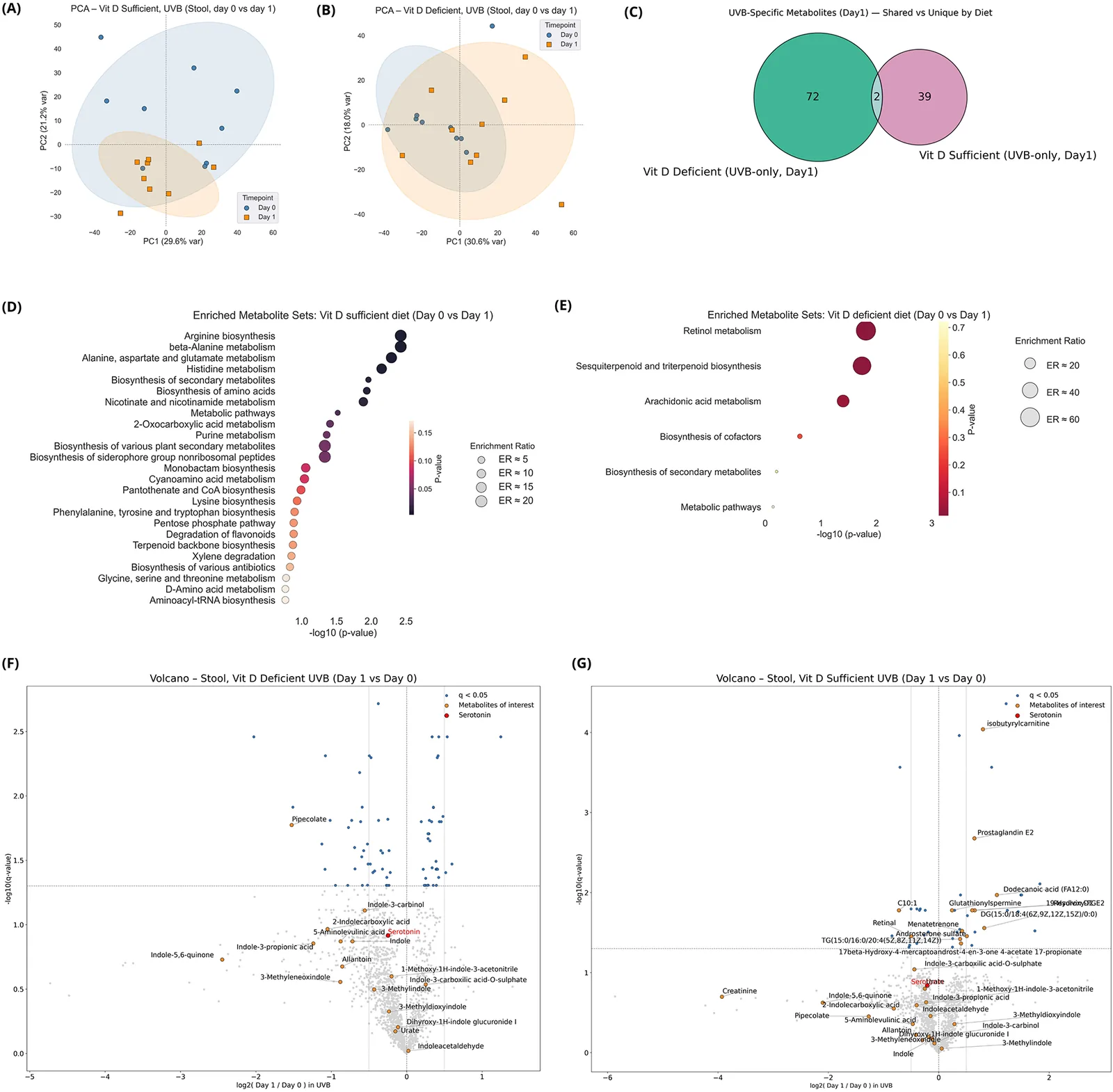
Acute Solius UVB light therapy induces limited, vitamin D–dependent remodeling of the stool metabolome at Day 1. **(A, B)** PCA of stool metabolomes at Day 0 (blue) vs. Day 1 (orange) following Solius UVB light therapy in vitamin D–sufficient **(A)** and vitamin D–deficient **(B)** mice. A significant temporal shift was detected only in vitamin D–sufficient mice (PERMANOVA: pseudo-F = 3.39, *p* = 0.011), whereas no significant separation was observed in vitamin D–deficient mice (pseudo-F = 1.87, *p* = 0.116). **(C)** Venn diagram of Solius-UVB-light-therapy-specific stool metabolites at Day 1 (FDR *q* < 0.05), defined as metabolites altered by Solius UVB light therapy but unchanged in matched controls, showing largely diet specific responses with minimal overlap between vitamin D–deficient and vitamin D–sufficient mice. **(D)** Pathway enrichment analysis of Solius-UVB-light-therapy-responsive metabolites at Day 1 in vitamin D–sufficient mice using MetaboAnalyst (Gut Microbiota + Host KEGG), revealing enrichment of amino-acid, nucleotide, and biosynthetic pathways. **(E)** Pathway enrichment of Solius-UVB-light-therapy-responsive metabolites at Day 1 in vitamin D-deficient mice, showing a smaller and more restricted set of enriched pathways. **(F, G)** Volcano plots illustrating Solius-UVB-light-therapy-induced stool metabolite changes at Day 1 relative to Day 0 in vitamin D–deficient **(F)** and vitamin D–sufficient **(G)** mice. Metabolites passing FDR correction (*q* < 0.05) are highlighted, with selected metabolites of interest annotated.

Principal component analysis (PCA) identified a shift over time in Solius-UVB-light-therapy-treated mice while control animals showed a modest drift of intestinal metabolites in both diets (Figures 4A, B; Supplementary Figures 3C, D). In agreement with these patterns, a PERMANOVA test showed a significant global metabolomic reorganization after a single Solius UVB light therapy in vitamin D-sufficient mice (pseudo*-F* = 3.39 and *p* = 0.011). In the case of vitamin D deficient mice, Solius UVB light therapy does not induce significant changes in the intestinal metabolome (pseudo*-F* = 1.87, *p* = 0.116). The non-exposed groups show that diet-dependent effects were small compared with Solius-UVB-light-therapy-driven changes, especially in the vitamin D sufficient group (Vit D-sufficient control: *p* = 0.033; Vit D-deficient control: *p* = 0.064, Supplementary Figure 3C and 3D).

To focus on the metabolites altered by Solius UVB light therapy, we excluded those that also exhibited changes in untreated-control mice. This process identified 72 metabolites that were altered exclusively in the vitamin D deficient mice and 39 metabolites that changed solely in the vitamin D sufficient mice, with only two metabolites (Mycinamicin I and 1-(1-Methoxy-1-methylethyl)-4-methylbenzene) being common to both groups (Figure 4C). These results suggest that the acute intestinal metabolic response to Solius UVB light therapy depends on vitamin D status.

Pathway enrichment analysis (MetaboAnalyst 6.0, Gut Bacteria and Host KEGG library) highlighted the co-remodeling of host-microbe systems (Figures 4D and 4E). In vitamin D-deficient mice (Figure 4F), most Solius-UVB-light-therapy-responding metabolites decreased, and in particular the compounds associated with amino acid/nitrogen metabolism, including arginine, pipecolate and 5-aminolevulinic acid. These decreases were associated with lower amounts of urate and allantoin, consistent with purine turnover and antioxidant buffering. In addition, levels of indole-related aromatic carbons also decreased, indicating a reduction in the microbial tryptophan catabolism, even though levels of classic indoles such as indole-3-propionic acid did not show significant changes (Figure 4F).

Vitamin D-sufficient mice displayed a more varied and adaptive metabolomic profile (Figure 4G). Acute Solius UVB light therapy was linked to subtle shifts in lipid- and eicosanoid-metabolic pathways: including arachidonate-containing species (e.g., TG(15:0/16:0/20:4(5Z,8Z,11Z,14Z))) and prostaglandin derivatives (prostaglandin E2, 19-Hydroxy-PGE2), as well as steroid and fat-soluble-vitamin metabolites (androsterone sulfate, menatetrenone). Some selected redox—and energy-metabolism markers (i.e., glutathionylspermine; isobutyrylcarnitine) also showed variable changes, but the effect of these changes was not clear. There was no change seen in levels of any SCFA at Day 1, in contrast to observations after 14 days of repeated Solius UVB light therapy.

In contrast to the robust metabolic remodeling observed at Day 14, acute Solius UVB light therapy elicited only modest and diet-dependent metabolic changes. These findings suggest that the dominant effects of Solius UVB light therapy on the gut metabolome emerge progressively with repeated exposures.

### 3.3 Serum metabolomes exhibit targeted cumulative responses to Solius light UVB therapy

Because Solius UVB light therapy produced substantial metabolic remodeling within the colonic lumen, we next examined whether these effects extended systemically. To address this, we analyzed serum metabolomic profiles following both acute (Day 1) and repeated (Day 14) UVB exposure. The analyses were not paired since the serum was collected at euthanasia. This limitation prevented within-animal longitudinal comparisons pre- to post Solius UVB light therapy. This approach did however enable the discovery of timepoint-specific, systemic metabolic signatures associated with Solius UVB light therapy.

#### 3.3.1 Repeated (Day 14) UVB exposure leads to systemic metabolomic modulation shaped by initial vitamin D status

**Figure 5:**
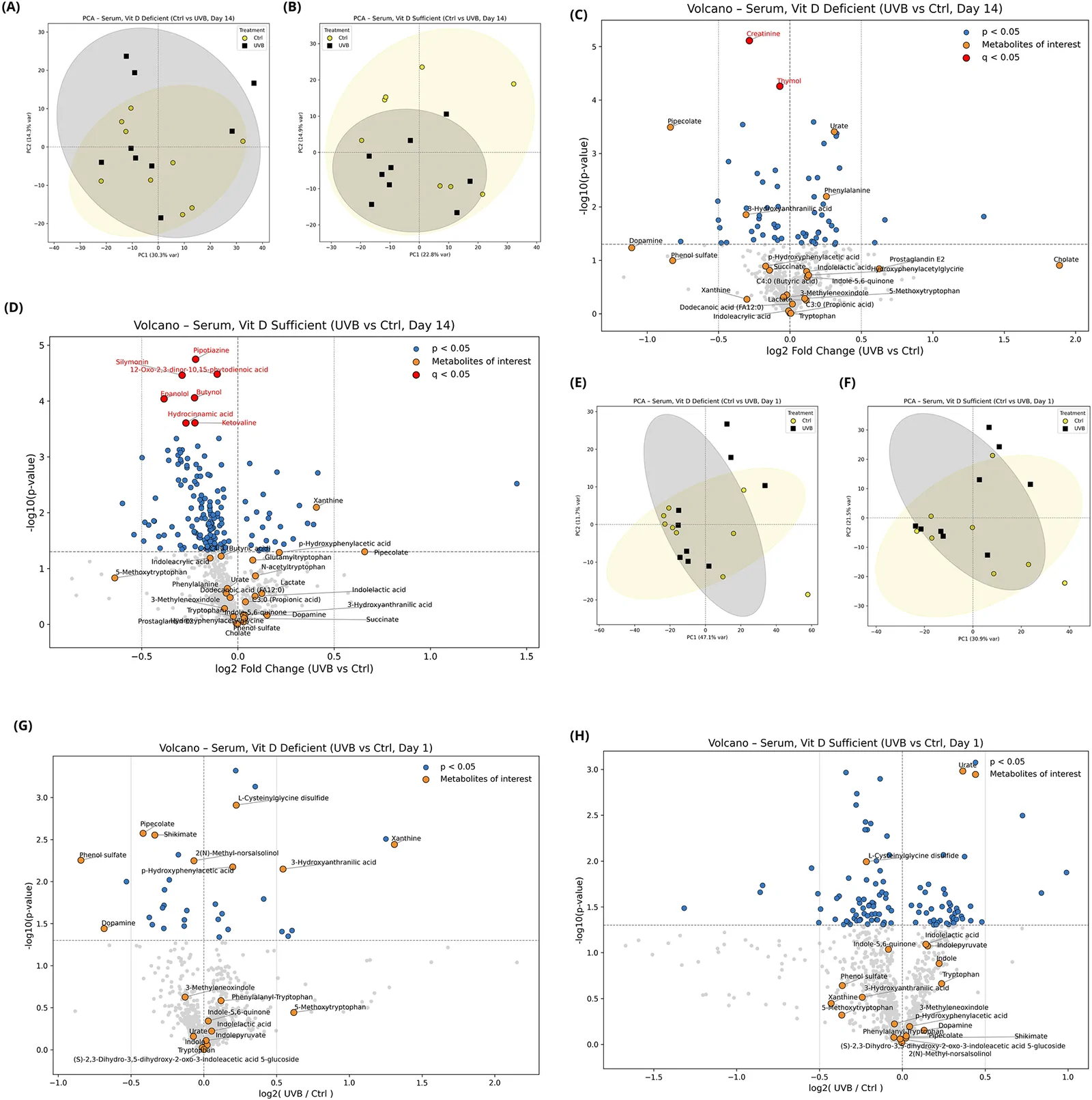
Solius UVB light therapy induces targeted rather than global changes in circulating metabolites, with effects shaped by vitamin D status**. (A, B)** PCA of serum profiles at **Day 14** in vitamin-D–deficient **(A)** and vitamin-D–sufficient **(B)** mice. As at Day 1, groups showed overlapping distributions with no robust global treatment effect (PERMANOVA: Vit-D sufficient *p* = 0.064; Vit-D deficient *p* = 0.292). **(C, D)** Volcano plots for **Day 14** in vitamin-D–deficient **(C)** and vitamin-D–sufficient **(D)** mice. Selected metabolites passing FDR correction or showing consistent Solius-UVB-light-therapy-associated trends are annotated. Signals included metabolites linked to amino-acid metabolism, purine/redox pathways, and tryptophan–kynurenine signaling, indicating focused systemic modulation despite limited global restructuring. **(E, F)** PCA of serum metabolomic profiles at **Day 1** in vitamin-D–deficient **(E)** and vitamin-D–sufficient **(F)** mice. Points represent individual animals (Ctrl = yellow, Solius UVB light therapy = black); ellipses show 95% confidence intervals. No global treatment effect was detected (PERMANOVA: Vit-D sufficient *p* = 0.329; Vit-D deficient *p* = 0.637). **(G, H)** Volcano plots for **Day 1**, showing log2(Solius UVB light therapy/Ctrl) vs. –log10(p) for vitamin-D–deficient **(G)** and vitamin-D–sufficient **(H)** mice. Blue points indicate nominal associations (*p* < 0.05), and red labels highlight biologically relevant metabolites discussed in the text. Although no metabolites passed FDR correction, trend-level changes involved purine/redox intermediates, microbial aromatics, and tryptophan- and neuroactive-related metabolites.

Six exposures to Solius UVB light therapy (Day 14) had little influence on the serum metabolome. Correspondingly, PCA also showed overlapping clusters between treated and untreated groups (Figures 5A, B). PERMANOVA analysis indicated that there was no clear effect of treatment (Vit-D deficient: pseudo*-F* = 0.0384, *p* = 0.292; Vit-D sufficient: pseudo*-F* = 0.0922, *p* = 0.064).

Although a global metabolic restructuring was not detected, some Solius-UVB-light-therapy-induced metabolites were found in particular pathways (Figures 5C, D). In vitamin D-deficient mice (Figure 5C), two metabolites passed FDR correction: creatinine (decreased with Solius UVB light therapy) and thymol (considered a xenobiotic). Further minor changes in the contents of pipecolate, urate, 3-hydroxyanthranilic acid and p-hydroxyphenyllactic acid suggested subtle regulation of nitrogen and redox metabolism.

In contrast, mice on a vitamin D sufficient diet exhibited relatively little change in serum metabolites following repeated Solius UVB light therapy, and assessed at Day 14 (Figure 5D). The volcano plot in Figure 5D highlights the few metabolites that reached significance (*p* < 0.05) and remained significant after false discovery rate correction. The observed responses included mild fluctuations in amino acid-related metabolites, including nucleotide- and purine-associated compounds (e.g., xanthine- and urate-related species). In particular, several tryptophan metabolites showed some direction of effect but were not significant after correction. These findings indicate that, in vitamin D sufficient mice, there are measurable but quantitatively restricted systemic metabolic responses to Solius UVB light therapy that parallel a more substantially buffered serum metabolome, as compared with the pronounced effects seen in the stool at this time point.

#### 3.3.2 Acute (Day 1) Solius UVB light therapy induces modest, systemic changes with minimal global remodeling

On Day 1, PCA of serum metabolites did not yield clear clustering between Solius-UVB-light-therapy-treated and control mice (Figures 5E, F), while PERMANOVA confirmed the lack of a global treatment effect (vitamin D-sufficient: pseudo*-F* = 0.0335, *p* = 0.329; vitamin D-deficient: pseudo*-F* = 0.0265, *p* = 0.637). Moreover, there were no serum metabolites of FDR significance (Figures 5G, H). We did however examine nominal trends (*p* < 0.05) to determine if Solius UVB light therapy did drive modest, yet biologically consistent changes. In vitamin D deficient mice, Solius UVB light therapy exposure was associated with changes in pipecolate, shikimate, xanthine and L-cysteinylglycine disulfide as well as the microbial-derived metabolites phenol sulfate and p-hydroxyphenylacetic acid. In mice fed a vitamin D deficient diet, Solius UVB light therapy also induced significant changes in tryptophan-related metabolites including 3-hydroxyanthranilic acid, 5-methoxytryptophan and phenylalanyl-tryptophan, whereas dopamine and the putative toxin 2(N)-methyl-norsalsolinol were decreased (Figure 5G).

Mice fed a vitamin D sufficient diet showed limited metabolic shifts, which included minimal changes in urate, several amino acids and the nucleotide-derived metabolite ADP-ribose2' as well as subtle changes in several microbial co-metabolites (Figure 5H). While the effects did not survive correction for multiple comparisons, they were in the same direction as those associated with stool, potentially indicating early systemic signaling.

Overall, Solius UVB light therapy induced metabolic changes were markedly more pronounced in the stool than in the serum. While selected metabolites exhibited consistent directional shifts between stool and serum, the limited systemic response suggests substantial physiological buffering, suggesting that most Solius UVB light therapy driven metabolic remodeling remains localized to the GI tract.

### 3.4 Predicted functional consequences of Solius UVB light therapy: IPA-inferred biological pathways

**Figure 6:**
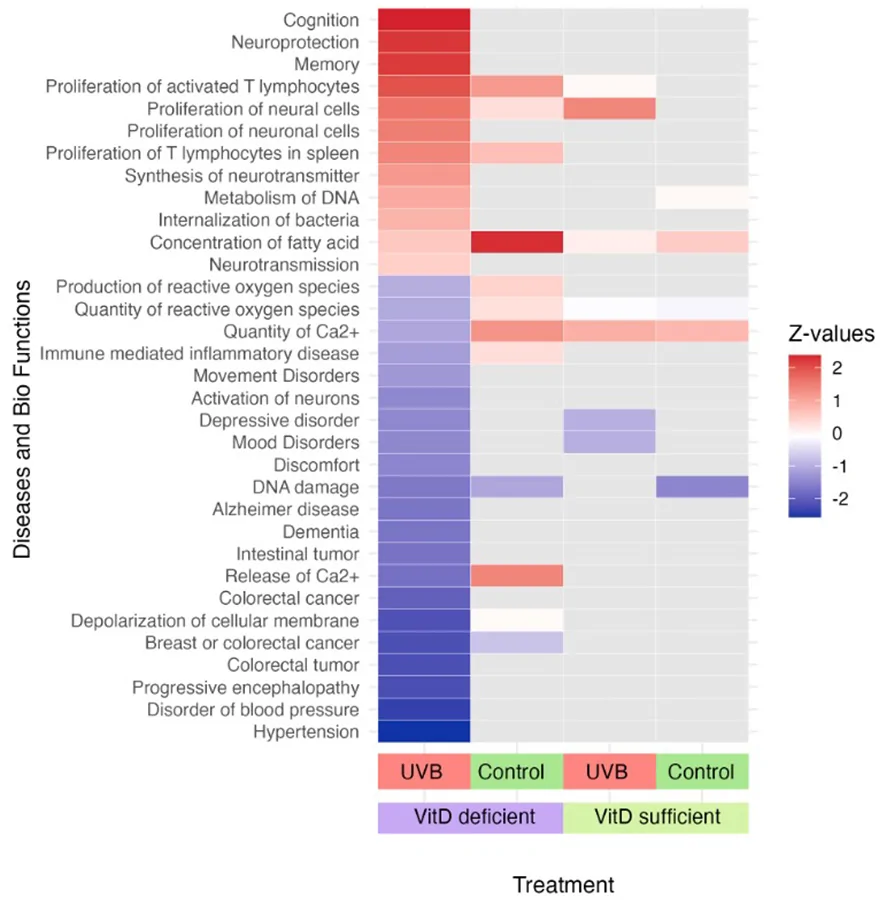
IPA-inferred biological functions associated with Solius-UVB-light-therapy-responsive stool metabolites (Day 14). Solius-UVB-light-therapy-specific metabolites (FDR *q* < 0.05; unchanged in controls) were analyzed using Ingenuity Pathway Analysis (IPA). The heatmap shows representative disease and biofunction categories with significant enrichment (overlap *p* < 0.05). Colors represent activation Z-scores, where red indicates predicted activation and blue indicates predicted inhibition. In vitamin-D–deficient mice, Solius UVB light therapy was associated with predicted suppression of inflammation- and pathology-related programs and activation of immune- and neuroprotective functions, whereas vitamin-D–sufficient mice showed more targeted regulatory effects. These predictions are hypothesis-generating and based on metabolite signatures rather than direct functional measurements.

As the stool metabolomes at Day 14 showed marked remodeling, we used Solius-UVB-light-therapy-responsive and control-responsive metabolites for inference of higher-order functional pathways based on Ingenuity Pathway Analysis (IPA) (Ingenuity Pathway Analysis | QIAGEN Digital Insights, 2026). IPA one by one overlays every metabolite on the well curated molecular networks and checks if a change in direction is consistent with previously known biological findings. From this analysis, two complementary metrics are generated: (i) an activation Z-score, which predicts the functional activation or inhibition, and (ii) an overlap *p*-value that measures enrichment of molecules pertaining to that function. Collectively, these metrics generate hypothesis-driving predictions which connect skin measurements of Solius UVB light therapy to downstream physiology in the gut and other tissue compartments (Figure 6).

In Solius-UVB-light-therapy-treated vitamin D-deficient mice, IPA inferred that treatment inhibited pathways related to intestinal tumor signaling and oxidative stress responses such as DNA damage cell membrane depolarization, calcium dyshomeostasis, and formation of ROS. Simultaneously, IPA suggested that Solius UVB light therapy treatment activated host functions linked to epithelial and mucosal support (including proliferation of activated T lymphocytes) as well as pathways involving neuroprotection, improved cognitive function and synthesis of neurotransmitter precursors. These inferences are consistent with the mucosal metabolic profile at Day 14, which revealed elevated amino acid and redox metabolism as well as an increase in indole/tryptophan-related products and SCFA-related pathways.

In contrast, control animals housed on vitamin D deficient diets showed only weak and inconsistent IPA signatures, suggesting little spontaneous drift (and supporting the view that these functional predictions are primarily determined by Solius UVB light therapy related changes in metabolites). Taken together, these findings indicate that Solius UVB light therapy of vitamin D deficient mice promotes a shift in host-microbiome interactions toward reduced inflammatory pressure, as well as up-regulation of neuroimmune signaling pathways.

As for vitamin D sufficient mice, IPA showed a reduction in neurobehavioral signaling changes with some modest changes observed in gut-associated disease pathways. Accordingly, vitamin D sufficiency appears to restrict Solius-UVB-light-therapy-derived effects to a smaller number of regulatory biofunctions, whereas vitamin D deficiency shows broader remodeling across intestinal and systemic functional networks. In general, our IPA corroborates our earlier metabolomic results by indicating that Solius-UVB-light-therapy-induced metabolic changes intersect at integrated gut-immune-neurobehavioral pathways. These inferences are only preliminary, but they do suggest that initial vitamin D status is a key modifier of the direction as well as magnitude of Solius-UVB-light-therapy-induced biological programs.

### 3.5 Integration of systemic (serum) and intestinal (stool) metabolomic profiles

To determine if the Solius-UVB-light-therapy-mediated metabolic reprogramming in the stool was accompanied by corresponding changes at a systemic level, we compared metabolite changes in the stool and serum (Table 1). At the stool level, Solius UVB light therapy was associated with a significant enrichment of several metabolite families involved in host-microbiome interactions. Among these were polyamine-related intermediates, fermentation products, and SCFA precursors, as well as metabolites associated with mucin turnover and bacterial cell wall remodeling. These changes suggest enhanced pathways related to epithelial barrier stability and mucus production, alongside microbial restructuring. At the same time, metabolites of the tryptophan/kynurenine and purine pathways showed consistent directional changes in stool, suggesting modulation of AhR-related and more general immunometabolic signaling networks.

**Table 1:** UVB-induced metabolite alterations across stool and serum under vitamin D–deficient and -sufficient dietary conditions.

Metabolite/class	Stool Vit D-def	Stool Vit D-suf	Serum Vit D-def	Serum Vit D-suf	Biological relevance
**Propionic acid (C3:0)**	↑	↑(trend)	≈	≈	SCFA, epithelial fuel, anti-inflammatory.
**Butyric acid (C4:0)**	↑	≈	≈	≈	SCFA, barrier integrity, goblet cells.
**Succinate**	↑	↑(trend)	≈	≈	Fermentation intermediate; microbiota cross-talk.
**Lactate**	↑(trend)	↑(trend)	≈	≈	Substrate for butyrate-producing bacteria.
**Isobutyrylcarnitine**	↑	↑	≈	≈	Reflects SCFA oxidation.
**Polyamines (e.g., N-acetylputrescine)**	↑	↑(trend)	≈	≈	Mucosal repair, epithelial renewal.
**Bile-acid derivatives**	↓(trend)	↓(trend)	≈	≈	Microbiota–bile axis; barrier signaling.
**Xanthine/xanthosine**	↑	↑	↓(trend)	↑(trend)	Purine-redox, host–microbiome co-metabolism.
**Pipecolate**	↑(trend)	↑ (trend)	↓(trend)	↑(trend)	Lysine/immune-linked; microbiome-derived.
**Tryptophan and** 3-Hydroxyanthranilic acid/**Kynurenine-related metabolites**	↑	↑(trend)	↓(trend)	≈	AHR/neuroimmune signaling.
**Dopamine-related intermediates**	↑	≈	↓(trend)	≈	Gut–brain axis candidate.

↑/↓ indicate directional change.

≈ indicates no change.

In contrast, bile acid-related metabolites were found to gradually fall in abundance within stool samples after Solius UVB light therapy, suggesting altered microbiota–bile acid dynamics within the intestinal lumen. Although these changes did not remain significant after adjusting for multiple comparisons (FDR-corrected), the concordant direction of change across multiple bile-acid molecules suggests a response to Solius UVB light therapy.

Changes in these metabolite classes in the serum, in response to Solius UVB light therapy were less common and generally of weaker effect. Most stool-directional metabolites showed no corresponding changes in the serum. However, for a smaller subset of metabolites (e.g., pipecolate and xanthine), the changes in stool was similar to that observed in serum. This indicates that some features of gut metabolic reprogramming may spread systematically.

Taken together, these analyses indicate that the effect of Solius UVB light therapy is predominantly characterized by coordinated and associated metabolic reprogramming in the stool/intestinal compartment, relating to SCFAs, fermentation intermediates, polyamines, mucin-and bacterial cell wall-related molecules as well as bile-acid derivatives and metabolites accompanying tryptophan and purine pathways. Only a subset of these changes was reflected in the serum.

### 3.6 Integrative interpretation of gut microbiota and metabolomic shifts

Having identified significant Solius UVB light therapy affected alterations in the gut microbiome and stool metabolome at day 14, we subsequently combined these two sets of data to evaluate if there were any closely-related associations between microbial taxa and metabolites. Because the most pronounced changes were observed in mice maintained on a vitamin D-deficient diet, subsequent analyses focused on this group.

**Figure 7:**
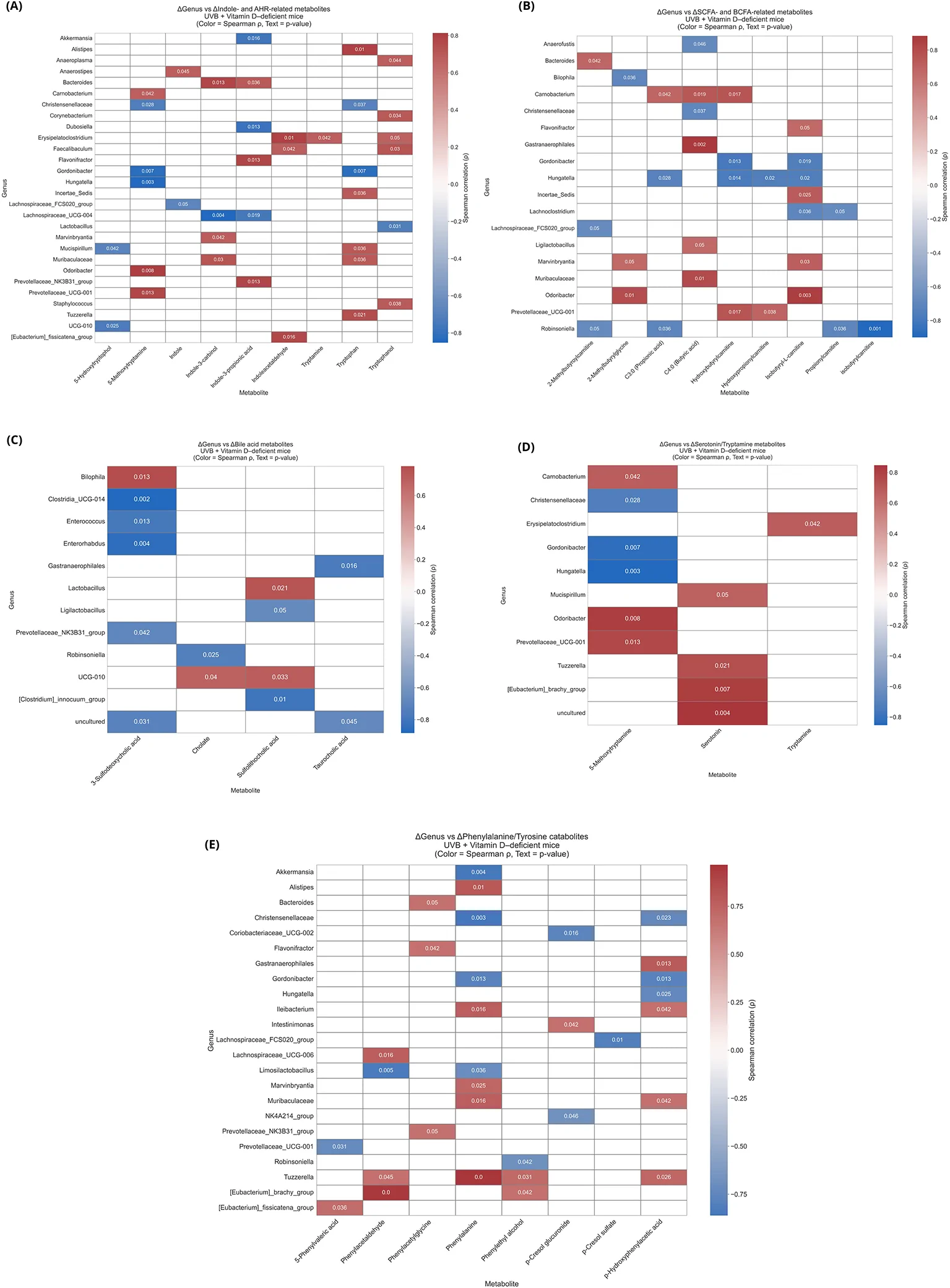
Microbiota–metabolome correlations at Day 14 (vitamin-D–deficient mice). Spearman correlations were computed using paired changes (ΔDay14–Day0) in genus abundance and stool metabolites. **(A)** Indole-related metabolites, **(B)** SCFAs and BCFA-related metabolites, **(C)** bile-acid–related metabolites, **(D)** serotonin/tryptamine metabolites, **(E)** phenylalanine/tyrosine catabolites. Heatmaps show correlation coefficients (ρ), with red = positive and blue = negative associations. Boxes indicate correlations with *p* < 0.05. None remained significant after FDR correction (*n* = 9); results are hypothesis-generating. Because microbiota data are compositional, associations should not be interpreted as causal but rather as candidates linking Solius-UVB-light-therapy-responsive taxa and metabolites. Summary figure created in BioRender. Vallance, B. (https://BioRender.com/dz8tdqb) is licensed under CC BY 4.0.

The change Δ was calculated (Day 14–Day 0) for both genus-level relative abundance and stool metabolite concentrations in each subject and associated changes were assessed using the Spearman correlation. Note that the microbial abundances are relative rather than absolute since they were estimated using 16S rRNA sequencing. As such, these analyses should be viewed as focused on finding biologically plausible relationships between microbes and metabolites rather than causative interactions. To facilitate biological interpretation, correlations were organized into pathway-focused heatmaps capturing distinct metabolic modules (Figures 7A–E).

Correlation analysis determined that specific tryptophan-derived metabolites were associated with directional metabolic changes of particular microbial taxa, rather than general associations for entire pathways (Figure 7A). Of the indole derivatives investigated, indole-3-propionic acid (IPA) presented the most characteristic and consistent correlation pattern. IPA was inversely correlated with the mucin-active genera *Akkermansia* and *Dubosiella*, which meant that higher abundance of these taxa was associated with reduced levels of this important epithelial-protective indole metabolite. Conversely, AhR-associated microbial-derived indoles, such as indoleacetaldehyde were positively associated with the *Faecalibaculum, Erysipelatoclostridium* and *Eubacterium_fissicatena* group*s*. Together, these metabolite-restricted signatures support the hypothesis that UVB-induced microbial remodeling impacts specific indole-producing pathways associated with epithelial and immune signaling, rather than providing broad modulation of tryptophan metabolism. Although not statistically significant after multiple-testing correction (*n* = 9), the consistency of these associations across independent metabolites is indicative of a potential relationship between Solius-UVB-light-therapy-mediated microbiota community shifts and increased production of AhR-active indoles.

Among the fermentation-related SCFA, we looked for associations with specific bacterial genera. Butyric acid had positive relationships with *Muribaculaceae, Gastranaerophilales, Ligilactobacillus* and *Carnobacterium* but showed inverse correlations with *Christensenellaceae* and *Anaerofustis* (Figure 7B). Propionic acid showed the same pattern of association (positively with *Carnobacterium* and negatively with *Hungatella*, and *Robinsoniella*) (Figure 7B). The SCFA-associated acyl-carnitines followed these same trends, suggesting that patterns of microbial fermentation are reflected in the subsequent metabolic processing by the host. These observations indicate restructuring of fermentation niches, rather than an expansion of just one SCFA producer.

Bile-acid related metabolites also showed similar concerted profiles (Figure 7C). The correlation patterns of the primary bile acid cholic acid were found to be divergent throughout taxa, whereas sulfated secondary bile acid compounds (i.e., 3-sulfodeoxycholic acid and sulfolithocholic acid) had a positive association with the bile-tolerant genera (e.g., *Bilophila*) and negative association with Clostridia UCG-014 and *Enterorhabdus* (Figure 7C). The negative associations between Taurocholic acid and *Gastranaerophilales* are consistent with bacterial deconjugation. While these observations are hypothesis-generating, they suggest that Solius-UVB-light-therapy-mediated changes in the microbiome also may affect bile acid metabolism across the gut-liver axis.

Correlation analysis revealed that changes in serotonin-related metabolites were positively associated with a limited subset of bacterial genera in Solius-UVB-light-therapy-treated vitamin D deficient mice (Figure 7D). Specifically, *Mucispirillum, Tuzzerella* and *[Eubacterium] brachy_*group exhibited significant positive correlations with serotonin-associated metabolites. No strong negative correlations with serotonin were observed, suggesting that Solius UVB light therapy preferentially aligns serotonin-associated changes with enrichment of specific taxa rather than broad antagonistic microbial effects. Importantly, these bacterial genera are not directly synthesizing serotonin. Instead, their associations likely represent an indirect modulation of host serotonin production or availability. This, moreover, is consistent with established mechanisms through which gut microbes affect the synthesis of serotonin primarily by modulating the activity of host enterochromaffin cells and the metabolic flux of tryptophan rather than through microbial serotonin biosynthesis (Reigstad et al., 2015; Yano et al., 2015).

Finally, aromatic amino-acid metabolism revealed distinct microbial signatures. Phenylalanine correlated positively with saccharolytic genera such as *Tuzzerella, Muribaculaceae, Marvinbryantia*, and *Ileibacterium*, but negatively with *Christensenellaceae, Akkermansia, Limosilactobacillus*, and *Gordonibacter* (Figure 7E). Downstream phenolic metabolites (p-cresol conjugates, phenylacetate intermediates and γ-valerolactone derivatives) grouped with bacterial taxa possessing proteolytic or polyphenol degrading activities, suggesting possible changes in the degradation of aromatic metabolites. While the associations were not significant when corrected for false discovery rate (FDR), they do appear to constitute biologically meaningful patterns.

Taken together, these integrative analyses suggest that Solius-UVB-light-therapy-induced restructuring of the gut microbiota occurs in a manner that correlates with metabolic pathways involved in indole/AHR signaling, SCFA fermentation, bile-acid metabolism and aromatic amino acid processing. These results should be considered preliminary due to compositional constraints and small sample size, yet they indicate mechanistic pathways that deserve to be pursued in dedicated follow-up studies.

## 4 Discussion

**Figure 8:**
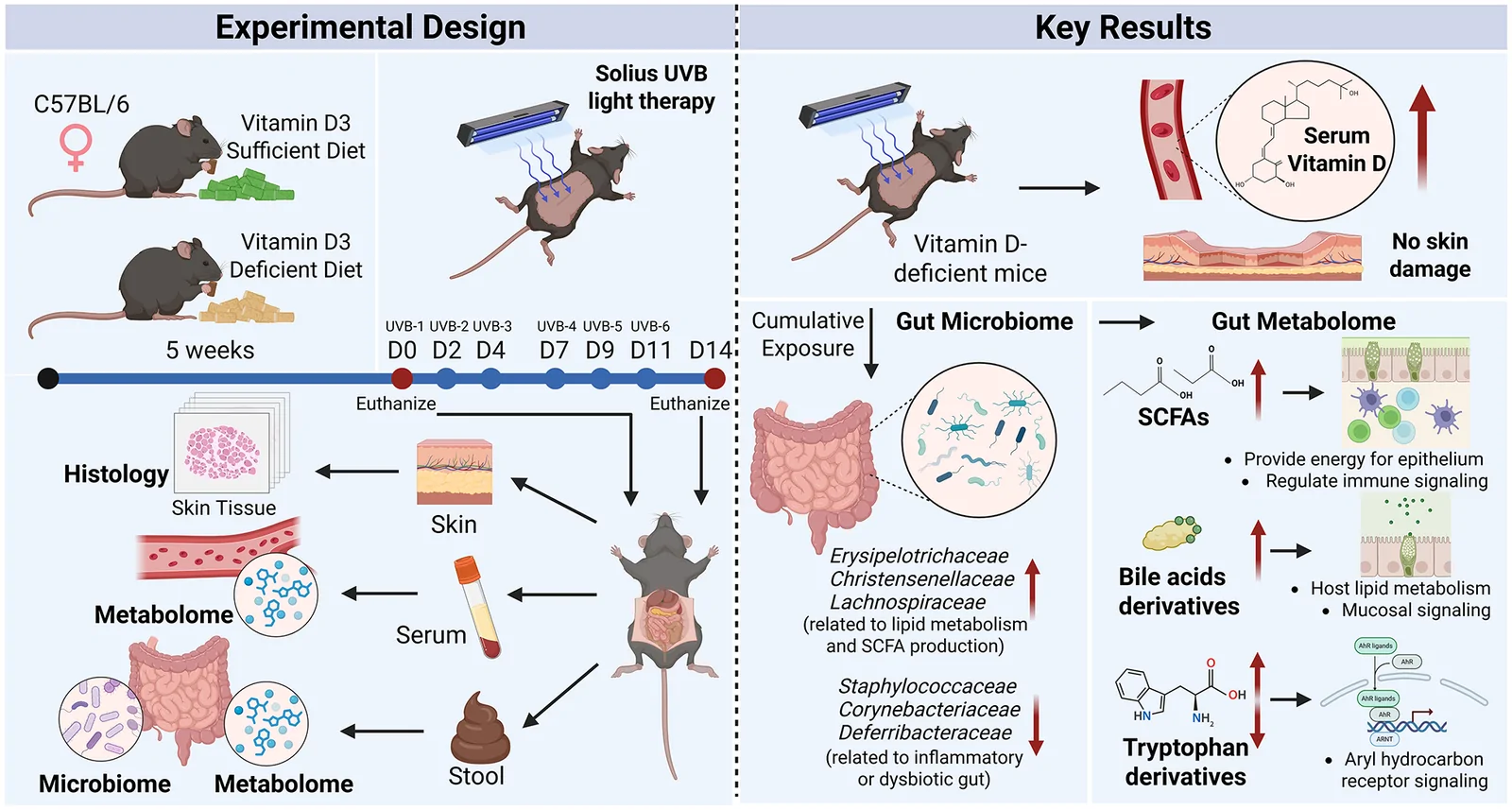
Summary of the experimental design and major findings following skin-targeted Solius UVB light therapy in mice. Female C57BL/6 mice were maintained on vitamin D_3_ sufficient or vitamin D_3_ deficient diets and exposed to Solius UVB light therapy. Skin, serum, and stool samples were collected for histology, microbiota, and metabolomic analyses. Solius UVB light therapy increased serum vitamin D levels without causing skin damage and reshaped the gut microbiota and metabolome, particularly in vitamin D-deficient mice. Repeated exposure enriched microbial taxa associated with metabolic health and SCFA production, while reducing inflammation-associated taxa. Metabolomic changes included increased SCFAs, bile acid derivatives, and tryptophan-related metabolites linked to epithelial function, immune regulation, and AhR signaling.

This exploratory study was designed to test a controlled skin-targeted Solius UVB light therapy protocol for both safety and biological effect. The Solius UVB light therapy increased serum levels of 25-hydroxyvitamin D_3_ after only one exposure and did not induce any overt skin damage. Repeated exposures led to physiological photoadaptation, supporting its tolerability and ability to preserve the skin barrier. It also enabled our exploration of downstream effects beyond the skin. The results demonstrate that Solius UVB light therapy skin exposure leads to a reshaping of the gut microbiota and stool metabolome (Figure 8), with baseline vitamin D status playing an important role in the magnitude of these effects, as well as in their temporal trajectory.

Importantly, as systemic levels of vitamin D rose after six Solius UVB light therapy exposures, metabolic changes associated with Solius UVB light therapy were more evident within the gut lumen as compared to the serum. This observation is consistent with evolving models of gut-skin communication. Another interesting observation from our study was that Solius UVB light therapy did not cause substantial temporal changes in microbial α-diversity, but instead shifted the structure of the microbial community over time. This finding supports the concept that Solius UVB light therapy alters gut microbiota proportion/makeup without causing any substantial loss of diversity. Interestingly, a previous murine study found that UVB exposure changed the entire microbiota community but did not change Chao1 **α** diversity (Ghaly et al., 2018).

Likewise, our previous study in humans that used controlled Solius UVB light therapy found differences in ß-diversity and specific taxa rather than a uniform increase in diversity (only Shannon index but not in Chao1), especially among participants who presented as vitamin D-insufficient (Bosman et al., 2019). These results collectively support the proposition that Solius UVB light therapy affects community structure as opposed to depletion at large scales, a feature consistent with adaptive ecological reconfiguration rather than dysbiosis (Hooks and O'Malley, 2017).

One of the trends we identified was that Solius UVB light therapy skin exposure altered the microbiota and metabolic pathways primarily in ways related to gut function. Among vitamin D-deficient mice, repeated Solius UVB light therapy resulted in the enrichment of families including *Christensenellaceae* and *Lachnospiraceae*, coupled with reduced representation of multiple taxa typically found within an inflamed gut (Figure 8). Importantly, *Christensenellaceae* expansion was only observed in Solius-UVB-light-therapy-exposed mice and not untreated controls, suggesting a treatment-specific response. This family has been consistently associated with metabolic health, leanness and resistance to obesity, suggesting that Solius UVB light therapy could expand microbial clusters linked with improved energetics (Waters and Ley, 2019). These changes in stool microbiota composition were mirrored in the stool metabolome at the same time point, with higher concentrations observed for SCFA, fermentation intermediates and SCFA-derived acyl-carnitines. SCFA, such as butyrate and propionate, are critically involved in supplying energy to epithelial cells as well as increasing production of mucins, that help promote immune tolerance (Parada Venegas et al., 2019). The enrichment of these metabolites suggests an increase in microbial fermentation and in the consumption of microbial metabolites as energy sources for the host epithelium. This is consistent with earlier observations that abundant levels of SCFA supports the maintenance of epithelial tight junctions and the suppression of inflammatory immune responses (Parada Venegas et al., 2019). These data favor the conclusion that exposure to Solius UVB light therapy initiates metabolic adaptation rather than stress-induced perturbations (Flint et al., 2012; Parada Venegas et al., 2019).

Chronic Solius UVB light therapy also affected a series of inter-related metabolic networks, under a vitamin D deficient diet. Alterations were observed in amino acid metabolism, purine turnover, redox pathways, and tryptophan-derived metabolites. Several of these metabolites have been shown to engage host immune pathways, especially those mediated by the AhR, a key player in epithelial integrity and immune homeostasis (Agus et al., 2018; Zelante et al., 2013). Although induction of intestinal AhR signaling by skin-targeted Solius UVB light therapy was not directly examined in this study, the observed enrichment of tryptophan-derived metabolites and indole-related molecules might impact intestinal mucosal immunity and barrier integrity. Serotonin is of particular interest, as it is a key neuropeptide that primarily originates in intestinal enteroendocrine cells (Appleton, 2018). There were opposing trends in serotonin between mice on a vitamin D sufficient diet, where levels rose toward significance, and deficient mice, in which serotonin levels decreased in stool samples. This discrepancy suggests that Solius UVB light therapy could potentially modulate the tryptophan–serotonin axis, depending on vitamin D status, and consequently impact gut-brain interactions as well as intestinal transit.

Pathway assessments from Ingenuity Pathway Analysis suggested that UVB exposure inhibited oxidative stress and tumor-related signaling, while also activating anti-inflammatory and neurobehavioral pathways, especially under conditions of vitamin D deficiency. It should be noted that, although these findings are preliminary, they are consistent with the metabolomics observations and indicate that Solius UVB light therapy can affect diverse pathways.

Serum responses were more modest, with fewer metabolites undergoing significant changes and even fewer remained significant after correction for multiple testing. Nevertheless, changes in pipecolate-, purines- and kynurenine-metabolism pathways were in line with involvement in microbiota-immune system communication (Cervenka et al., 2017). The limited matching between stool and serum suggests biological buffering and perhaps also that serum was not collected longitudinally from the same animals, unlike stool samples. These results suggest that most Solius-UVB-light-therapy-induced metabolism takes place in the gut, with only select signals reaching the circulation.

The strength of the response to UVB exposure appeared to strongly depend on the host's initial vitamin D status. Vitamin D-deficient mice displayed extended remodeling of the microbiota and higher metabolic reconfigurations than vitamin D sufficient mice. This is in line with previous studies showing that increases in vitamin D levels impact both the gut microbiota as well as mucosal immunity (Bashir et al., 2016; Ooi et al., 2012). Thus, the diverse responses detected in vitamin D-deficient mice exposed to UVB likely reflect the impact of rising vitamin D levels, while the more modest responses seen in vitamin D-sufficient mice likely reflect vitamin D independent effects of the UVB exposure.

Combining microbiota with metabolome data revealed several functional themes in exposed mice, including elevated SCFA production, indole/AhR signaling, bile acid metabolism, and aromatic amino acid degradation (Figure 8). While associations no longer reached significance after correction, the observed trends were in the expected direction based on biology and previously published work. Multiple potential mechanisms could explain these effects; in addition to Solius UVB light therapy directly raising circulating vitamin D levels, it may also influence stress responses and neuroendocrine signaling systems as well as immune tone, all of which affect the gut (Kühn et al., 2024; Slominski et al., 2024). While our study was not designed to unravel these pathways, the concordance across datasets indicates that Solius UVB light therapy is not a system disrupter but instead may act as a physiological regulator. Targeted metabolite assays, mechanistic microbiome studies, and animal models with modified vitamin D signaling will be necessary for further interrogation of this hypothesis.

This study does have limitations. Untargeted metabolomics yield only semi-quantitative estimates for certain classes of metabolites, and the pathway analysis tools make predictions but not direct functional measurements. Furthermore, lack of repeated longitudinal serum sampling from the same individuals limited sensitivity in detecting systemic changes over time. Lastly, results in mice might not completely apply to humans. Nonetheless, the integration of serial stool measurements with cross-sectional serum metabolomics and microbiome profiling provides a foundation to examine the interface of Solius UVB light therapy exposure and intestinal function.

In conclusion, Solius UVB light therapy was associated with metabolic environments dominated by increased microbial fermentation, modulation of tryptophan- and bile-acid-related pathways and reduced metabolic signatures of oxidative stress. These effects were even more marked in the context of vitamin D deficiency, suggesting that baseline vitamin D levels can modulate the response to Solius UVB light therapy. Our results support further exploration of Solius UVB light therapy as a physiological amplifier of host-microbial relationships and not just as a cutaneaous treatment.

## Data Availability

The raw data supporting the conclusions of this article will be made available by the authors, without undue reservation.
