# Supplementary material for: Skin-directed Solius UVB light therapy increases vitamin D levels and alters intestinal microbiome and metabolome homeostasis
Source: Front Microbiol. 2026 Aug 12;17:1850035. doi: 10.3389/fmicb.2026.1850035 (PMC13508600; doi:10.3389/fmicb.2026.1850035)
Supplement: Supplementary file 1 [file Data_Sheet_1.docx]

Supplementary Material

# 1 Supplementary Figures

Supplementary Figure 1 Heatmap showing **temporal changes in the relative abundance of bacterial taxa** across major phyla in **control (non-SNB-UVB–exposed) mice**, comparing **Day 0 and Day 14** under vitamin D–sufficient and vitamin D–deficient conditions. Differential abundance analysis was performed between Day 14 and Day 0 within each dietary group. Colors represent **log2 fold change (log2FC)**, with **red indicating enrichment** and **blue indicating depletion** at Day 14 relative to Day 0. Only taxa meeting the specified statistical significance threshold (P < 0.05) are shown. Taxa are grouped by phylum to highlight diet-dependent temporal shifts in microbial composition in the absence of SNB-UVB exposure.

Supplementary Figure 2 Stool metabolomic variability in control animals. Principal component analysis (PCA) plots show stool metabolomic profiles of control mice at baseline (Day 0; blue) and after 24 hours or day 14 (Day 1or Day 14; orange), stratified by vitamin D status, with vitamin D deficient mice shown in panels (A) and (C) and vitamin D sufficient mice shown in panels (B) and (D). Ellipses denote 95% confidence intervals for each timepoint. A significant 24-hour shift in stool metabolomic composition was observed in vitamin D sufficient control mice (PERMANOVA: pseudo-F = 2.08, p = 0.033), whereas vitamin D deficient controls showed a non-significant trend (pseudo-F = 2.03, p = 0.064). At Day 14, significant temporal separation was detected in both vitamin D deficient (pseudo-F = 2.39, p = 0.008) and vitamin D sufficient control mice (pseudo-F = 2.28, p = 0.005).
